# Assessment of air pollution toxicity in urban environments in Barcelona, Spain, using a mobile air-liquid interface system

**DOI:** 10.1007/s11356-026-38306-6

**Published:** 2026-10-21

**Authors:** Fulvio Amato, Maryam Pyambri, Sharon Ridolfo, Carmen Bedia, Marina Ricarte, Angeliki Karanasiou, Ainhoa Rodriguez, Xavier Querol, Andrés Alastuey, Melissa Faria, Sylvia Mansilla, Micol Introna, Ana T. Juárez-Facio, Sarah S. Steimer, José Portugal, Benjamin Piña, Karine Elihn

**Affiliations:** 1 Institute of Environmental Assessment and Water Research, CSIC, 08034 Barcelona, Spain; 2 Department of Environmental Science, Stockholm University, 11418 Stockholm, Sweden

**Keywords:** Ozone, Nanoparticles, PM_2.5_, Gaseous air pollutants, Harbor, Road traffic, ALI exposure

## Abstract

Toxicity of airborne particles and gases was analyzed using a mobile air-liquid interface (ALI) exposure system at a busy harbor and an urban background site in Barcelona, Spain. Toxic effects were assessed in human A549 cells exposed directly to urban air by measuring lactate dehydrogenase release. Simultaneously, air quality measurements were conducted to monitor particle and gas concentrations. At both sites, air pollution was highly influenced by transport emissions from marine or terrestrial sources. Partial least squares (PLS) analysis predicted up to 80% of ALI toxicity variance based on air quality parameters. The analysis revealed correlations between air toxicity and both gases and particles representing both primary and secondary air pollutants. Notably, gas phase pollutants, particularly ozone, were the main contributors to the observed toxicity. In addition to demonstrating the effectiveness of the ALI system for in situ assessment of the toxicity of ambient air pollutants, our study emphasizes the importance of combining ALI exposures with air quality measurements to identify the specific components responsible for harmful effects.

**Graphical Abstract:**

**Figure:**
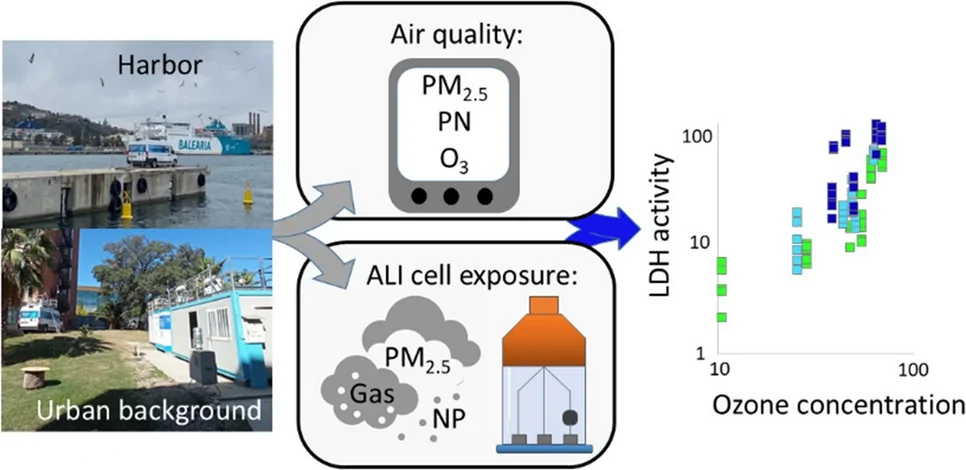

**Supplementary Information:**

The online version contains supplementary material available at https://doi.org/10.1007/s11356-026-38306-6.

## Introduction

Air pollution is the single largest environmental health risk in Europe and a major cause of premature death and disease worldwide (WHO Regional Office for Europe 2013; Kwon et al. 2020; Schraufnagel 2020; Kelly and Fussell 2020; Portugal et al. 2024). Despite the recognized importance of air pollutants’ impact on global health, identifying the specific pollutants responsible for these negative effects remains challenging (Kumar et al. 2013). While identifying particulate and gas-phase pollutants in the air is currently well established, their toxic potential is still not fully characterized (Portugal et al. 2024). Unlike aquatic pollution, there is no established methodology to assess the in situ impact of air pollution, neither through in vitro tests nor by monitoring recognized sentinel species. Standard in vivo toxicological tests, such as the Organisation for Economic Co-operation and Development (OECD) 28-day (subacute) inhalation toxicity study (OECD test 412) or the Fixed Concentration Procedure (OECD test 433), are cumbersome, time-consuming, and more suited for assessing the toxicity of individual substances rather than for the study of real-world complex mixtures. Moreover, these tests involve the use of experimental animals, such as rodents. In this context, the use of non-animal alternative methodologies (NAMs) offers a good approach to reduce the costs and time associated with toxicological assessments.

To some extent, NAM assays developed for toxicity testing under submerged conditions may also be applied to assess airborne toxicity (Sheesley et al. 2004; Cavanagh et al. 2009; Steenhof et al. 2011). These assays typically utilize cultured cells, animal tissues, or embryos at life stages where animal welfare restrictions do not apply (Fröhlich and Salar-Behzadi 2014; Rothen-Rutishauser et al. 2023; Hakkarainen et al. 2023; Portugal et al. 2024; Ricarte et al. 2025). These assays enable the testing of toxic effects across multiple cellular targets, or even the entire organism in the case of embryos; however, they have two significant drawbacks. First, they rely on collecting airborne particles using collection devices and filters, followed by resuspension of the collected material. Filters and filter collectors are designed to collect atmospheric particulate matter (PM) of various sizes (e.g., < 2.5 µm, PM_2.5_), whereas sorption material can be used to collect gaseous pollutants (Gioda et al. 2022; Nováková et al. 2022; Portugal et al. 2024; Fu et al. 2024). However, the removal or extraction processes may change the structure and toxicity of the original pollutants (Portugal et al. 2024). Second, collecting airborne material for subsequent toxicological and/or chemical analyses can take days or even weeks, potentially resulting in the loss of valuable temporal information that could reveal relevant correlations between sources, pollutants, and toxic effects. Long sampling periods may also result in partial transformations of the collected material. Air-liquid interface (ALI) exposure systems have been developed to overcome these limitations (Kim et al. 2013; Upadhyay and Palmberg 2018; Zavala et al. 2020; Grytting et al. 2024). ALI systems replicate the exposure of lung epithelia to airborne emissions by growing epithelial cells in a setup that allows their direct contact with an aerosol flow. This enables airborne particles to deposit directly onto semi-humid apical cell surfaces, resembling in vivo particle deposits on the lung surface. Furthermore, ALI systems are the only method that can assess the toxicity of both particle- and gas-phase pollutants, as cells are exposed to both phases simultaneously (Upadhyay and Palmberg 2018; Dilger et al. 2023; Hakkarainen et al. 2023; Grytting et al. 2024).

In this study, we used a mobile ALI system to test the toxicity of air pollution outdoors. The ALI system contained cultured human lung cells (A549) that were exposed to a flow of real-world particles and gases at actual outdoor locations, such as harbor and urban sites in Barcelona, Spain. The ALI system provides an experimental model for investigating cellular responses to direct exposure to airborne pollutants under air-liquid interface conditions. The system was particularly designed for the in situ monitoring of local air pollution sources outdoors (Juárez-Facio et al. 2024). The outdoor toxicity tests were synchronized with online air quality measurements, providing an accurate determination of the concentrations of air pollutants to which the lung cells were exposed. The combination of high-resolution temporal and spatial data offered a unique opportunity to accurately track the toxicity associated with air pollution. Furthermore, separated chambers containing A549 cells were exposed to filtered or unfiltered air, which allowed us to differentiate between the toxic effects of gas-phase and particulate pollutants. A549 cells were selected as a well-characterized human alveolar epithelial cell line that provides a robust and reproducible model for investigating acute cellular responses to complex ambient air exposures under air-liquid interface conditions. The use of A549 cells for short-term acute exposure studies under ALI conditions has also been described previously (Juárez-Facio et al. 2024). LDH release was selected as the primary cytotoxicity endpoint because loss of plasma membrane integrity is a well-established indicator of acute cytotoxicity and provides a quantitative measure of overt cellular damage. This endpoint also facilitates comparison with previous studies investigating the toxicity of airborne particles and complex air pollution mixtures. By combining simultaneous toxicity and air quality measurements, we aimed to identify the key contributors to air pollutant toxicity.

## Materials and methods

### Air–liquid interface (ALI) system

A mobile air-liquid interface (ALI) exposure system (Juárez-Facio et al. 2024) was used to assess the toxicity of airborne nanoparticles (Impactor cut-off 450 nm; hereafter referred to as nanoparticles, NPs, custom-made impactor), PM_2.5_ (particulate matter < 2.5 µm, SCC 1.062 Triplex cyclone, BGI by Mesa Labs, USA), as well as the gas phase on cell cultures. The ALI system consisted of six separate exposure chambers, each containing inserts with cultured lung cells. Air temperature within the system was maintained at 37 °C. A combination of a dryer (MD-110-12S-4, Perma Pure, Lakewood, USA) and a humidifier (MH-110-12S-4; Perma Pure, Lakewood, USA) ensured that the air reaching the cells had a relative humidity greater than 90%, independent of the outdoor conditions. This setup granted stable temperature (37 °C) and humidity (> 90%RH) inside the ALI system, even when ambient outdoor levels were lower. In the ALI system, cells were directly exposed to a flow of air (214 mL/min) in a way that airborne particles and pollutants settle on the cells (electrostatic deposition ± 1 kV), thereby simulating human inhalation. These conditions were selected based on our previous studies and ensure that the cells are not harmed under non-exposure conditions and promote optimal particle deposition (Juárez-Facio et al. 2024). Losses of particles to the walls in the ALI system (before the exposure chambers) were only 3.5% or less for > 30 nm-particles according to calculations using the particle loss calculator (Juárez-Facio et al. 2024). Furthermore, the aerosol flow and electrostatic field were carefully balanced to deposit particles only onto the cell surface and not onto the plastic walls of the cell inserts. Therefore, the particles and gases that reach the cells can be considered as a good representation of the outdoor aerosol at the harbor and urban background sites. Four of the chambers exposed the cells to aerosol, while the remaining two chambers used filtered, PM-free air to assess the toxicity of the gas phase (inorganic gaseous pollutants and volatile organic compounds). Each exposure chamber contained culture medium that nourished the cells through the semi-permeable membrane of the cell insert. Cell cultures seeded, handled, and transported in parallel to the exposed cultures, but without undergoing exposure, were used as control (2 controls for each exposure experiment).

### Outdoor locations for the ALI toxicity testing and air quality measurements

The ALI system was installed in a van to enable on-site toxicity testing of outdoor air at two locations in Barcelona (see supporting information, Figure SI1). This setup allowed ambient air to be drawn directly into the system at each measurement site. Two toxicity testing campaigns were conducted near the Barcelona harbor (HA) from January 24 to 26, 2023, and from January 31 to February 2, 2023. The HA site is located downwind of cruise and shipping emissions and as far as possible from road traffic. Air quality measurements were performed simultaneously using the same van equipped with air quality monitoring instruments at the HA site. A third toxicity testing campaign was conducted at an urban background site (BKG, Palau Reial) from September 29 to October 6, 2022. The BKG site is situated within the University of Barcelona campus, 220 m from one of the city’s main roads, Diagonal Avenue. The same ALI exposure system was used at both sites, located 6 km apart, in the city of Barcelona, Spain. A fixed air quality laboratory at the BKG site was used to monitor air quality parameters. The monitoring was done simultaneously with the BKG site toxicity tests. Ambient air temperature ranged from 9 to 23 °C, and relative humidity varied between 43 and 91%. No precipitation occurred during the measurement periods. The main characteristics of the campaigns including site location, date, and examined particle fraction are detailed in Table SI1, and photos of the measurement sites are shown in Figure SI1 of the supporting information.

### Cell culture and exposure

Human A549 cells (type II alveolar epithelial cell line, ATCC collection) were cultured at 37 °C in a humidified atmosphere with 5% CO_2_ in DMEM medium (Invitrogen) containing 2 mM L-glutamine and 1 mM pyruvate, 10% fetal bovine serum (Invitrogen), and 100 U/mL penicillin and 100 µg/mL streptomycin. Cells were subcultured when reaching about 80% confluence. To prepare for the exposure, cells were trypsinized, seeded at a density of 2.5 × 10^4 ^cells/well onto cell culture inserts (Falcon insert, 0.4 µm pore size, transparent PET membrane) with a semipermeable membrane at the bottom, placed in 6-well plates, and allowed to attach for 24 h before exposure in the ALI system. For transporting cells to the exposure site, the medium from the apical side was removed, and the medium in the basal chamber was supplemented with 25 mM HEPES (pH 7.4). Exposures were conducted for 2 h. Two separate exposure experiments were typically performed each day, one in the morning and one around midday. Two inserts were exposed to gaseous emissions (filtered air without particles, Headline filters, In-line filter, Grade DIF-LN40, particle removal efficiency of > 99.9999% for 0.1 µm, filters contain no gas adsorbents and are kept at 37 °C to minimize potential losses of reactive and semi-volatile compounds), while the others were exposed to the total aerosol (including both gases and particles). At the same time as the ALI toxicity testing and at the same places, the air quality was monitored. After the 2-h exposure, cells were incubated at 37 °C and 5% CO_2_ for 24 h before analysis. Control cells, processed alongside the exposed cultures, but left unexposed, were included for all campaigns and sites (eight samples for the background site, six samples for each of the harbor site campaigns).

### Cell toxicity assays

To assess cell viability following exposure to environmental pollutants in the ALI system, we determined the extracellular lactate dehydrogenase (LDH) activity, which measures the cell membrane damage by detecting LDH release into the culture media. LDH activity was quantified using a previously described microplate protocol (Diamantino et al. 2001; Kumar et al. 2018), where the decrease in absorbance at 340 nm, due to NADH oxidation, was measured with an Infinite M Plex (Tecan) microplate reader. LDH activity was expressed in Units (U)/1000 cells (1 U = 1 µmol substrate hydrolyzed per minute). Cells growing in an insert were incubated with 0.1% Triton as positive control.

### On-line air quality measurements

Air quality measurements were carried out using a mobile laboratory van at the HA site and a fixed monitoring station from the local network at the BKG site. Air quality parameters were measured simultaneously with the toxicity tests at each site (2 h), in order to determine the concentrations that the cell cultures were exposed to. Both monitoring sites were equipped with reference instruments for measuring ozone (O_3_, Thermo Scientific 49I; Thermo Fisher Scientific, Waltham, MA, USA), sulfur dioxide (SO_2_, Thermo Scientific 43i; Thermo Fisher Scientific, Waltham, MA, USA), and nitrogen oxides (NO, NO_2_, NOx, Thermo Scientific 42i; Thermo Fisher Scientific, Waltham, MA, USA). Black carbon (BC) concentrations were measured using a multi-wavelength dual-spot aethalometer (AE33; Magee Scientific, Berkeley, CA, USA). Particle number (PN) concentration was measured with a Scanning Mobility Particle Sizer (SMPS 3080; TSI Inc., Shoreview, MN, USA) coupled to a Condensation Particle Counter (CPC 3772; TSI Inc., Shoreview, MN, USA), covering a size range from 10 to 480 nm. Mass concentrations of particulate matter (PM_10_, PM_2.5_, PM_1_) were measured using an optical particle counter (GRIMM 1.180; GRIMM Aerosol Technik, Ainring, Germany). Measurement of PM_2.5_ and nanoparticle mass concentrations using electrical low-pressure impactor (ELPI, Dekati, Finland) determined the dose in ALI exposure experiments. It should be noted that volatile organic compounds were not monitored in this study.

### Data analysis

Statistical analyses were used to identify chemical compounds and physical properties associated with toxicological responses. Differences between exposed and control samples were analyzed using ANOVA plus Tukey’s post hoc analysis. Both uni- and multivariate approaches were employed to find connections between concentrations of air pollutants and LDH activity. All analyses were conducted in the R environment (R Studio 2022.07.1) using the following packages: *stats* for ANOVA/Tukey’s, *readxl* for data import, *ggplots* and *colorspace* for heatmaps with hierarchical clustering; *graphs*, *psych*, and *corrgram* for correlation matrices; and the *plsreg1* function from the *plsdepot* for partial least squares (PLS) regression. PLS1 analysis was performed using LDH values (logarithmic values) as predicted variable and the available emission dataset (PM_1_, PM_2.5_, PM_10_, black carbon, ozone, SO_2_, NO, NO_2_, NOx, PN), also as logarithm-transformed, as predictive variables. The whole dataset is shown in the Supplementary Table SI2. Leave-one-out cross validation indicated three components as the most adequate number, based on the respective Q2 values (see the PLS section in Supplementary material). Parametric correlation (Pearson’s) was the primary statistical method, with false discovery rate (fdr) post hoc analysis applied when appropriate. Since most datasets did not follow a normal distribution, logarithmic transformations were used for parametric statistics.

## Results

### Cell toxicity of air pollutants at harbor and urban background sites

**Fig. 1:**
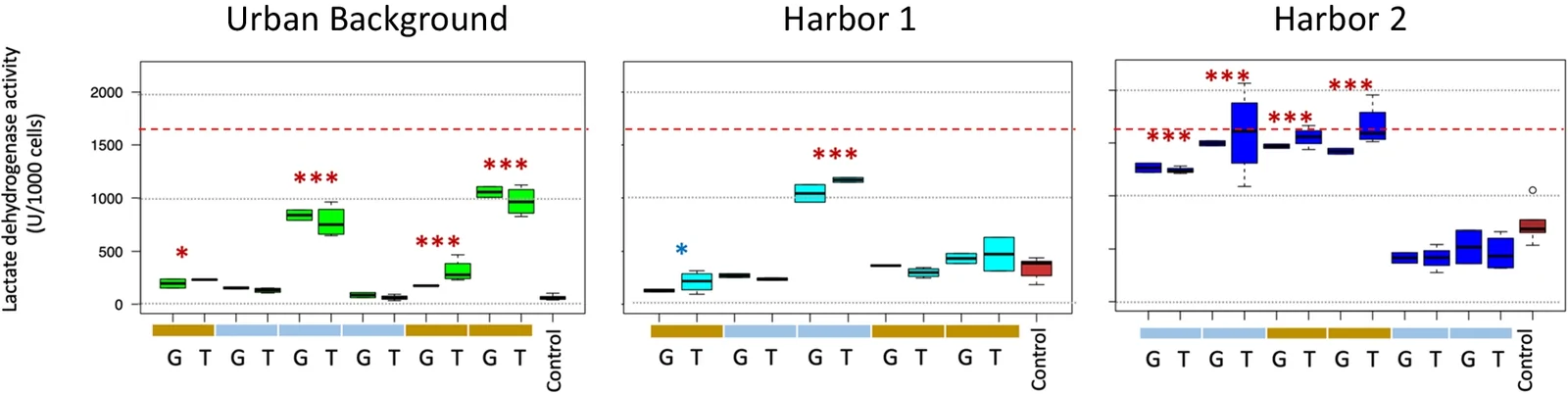
LDH activity values for the different sampling events from the three campaigns (one at the background site and two at the harbor site), shown in separate panels with identical Y-axis scales. Boxes represent the interquartile range (second and third quartiles), whiskers indicate the full value range, and the thick black line denotes the median. A single outlier is shown as an open circle in the Harbor 2 panel. For each exposure experiment, results are presented separately for filtered (gas-phase only, labelled G) and total aerosol (PM + gases, labelled T) samples on the bottom axis. Colored bars at the bottom axis indicate the particle cut-off size used in each campaign: orange for the 2.5 µm cut-off and blue for the NP cut-off (see supporting information Table SI1 for details). Box colors distinguish the three campaigns: green (urban background), light blue (harbor 1), and dark blue (harbor 2). Control values (from unexposed cells handled in the same way as the exposed ones) are shown for each site (red boxes). Asterisks indicate sampling events with LDH distributions significantly different from their corresponding controls (ANOVA + Tukey’s post hoc analysis; *p < 0.05; **p < 0.01; ***p < 0.001). Red and blue asterisks indicate distributions above and below control values, respectively. For statistical analysis, filtered and unfiltered samples from a same sampling event were combined to increase statistical power. The dashed red line represents the positive control (triton) at 1600 U/1000 cells

Cell toxicity caused by air pollutants, including NPs, PM_2.5_, and the gas-phase pollutants, was assessed by measuring LDH release from exposed cells. LDH activity values differed significantly both between locations and among campaigns conducted at the same site (Fig. 1). The figure presents results from both exposed samples and unexposed controls in each panel. As no significant differences were observed between filtered samples (gas-phase exposure only, labelled G in Fig. 1) and unfiltered samples (the total aerosol, i.e., co-exposure to both particles and gases, labelled T in Fig. 1) within the same exposure experiment, these values were combined for statistical comparison between sampling events and their respective controls.

Nine of the 17 sampling events showed LDH values significantly higher than the corresponding controls (red asterisks in Fig. 1), whereas one of them showed values below control levels (blue asterisk), which we attribute to experimental variability. The highest LDH activity was observed during the second HA campaign (Fig. 1). No influence of particle size (NP or PM_2.5_) in the toxicity was observed (PM_2.5_ = orange, and NP = blue bars at the bottom of the Y-axis in Fig. 1). Overall, these results indicate clear damage to the exposed cells in many of the sampling events both at the harbor and background sites. Under the conditions of this study, the absence of differences between filtered (G) and unfiltered exposures (T) suggests that gas-phase pollutants likely played a significant role in the observed toxic responses.

### Air quality measurements

Nanoparticle concentrations were relatively similar across the three campaigns, ranging from 9800 to 21,100 particles/cm^3^ at the BKG and from 10,500 to 17,900 particles/cm^3^ at the HA site. This suggests that the concentrations in the harbor were not notably higher compared to the background site. A similar trend was observed for O_3_ concentrations, while SO_2_, NO_2_, NO_x_, and BC concentrations were higher at the BKG site. In contrast, the highest PM concentrations (PM_1_, PM_2.5_, and PM_10_) were observed at the HA site (20.7, 24.5, and 88.8 µg/m^3^), particularly during the second campaign.

**Fig. 2:**
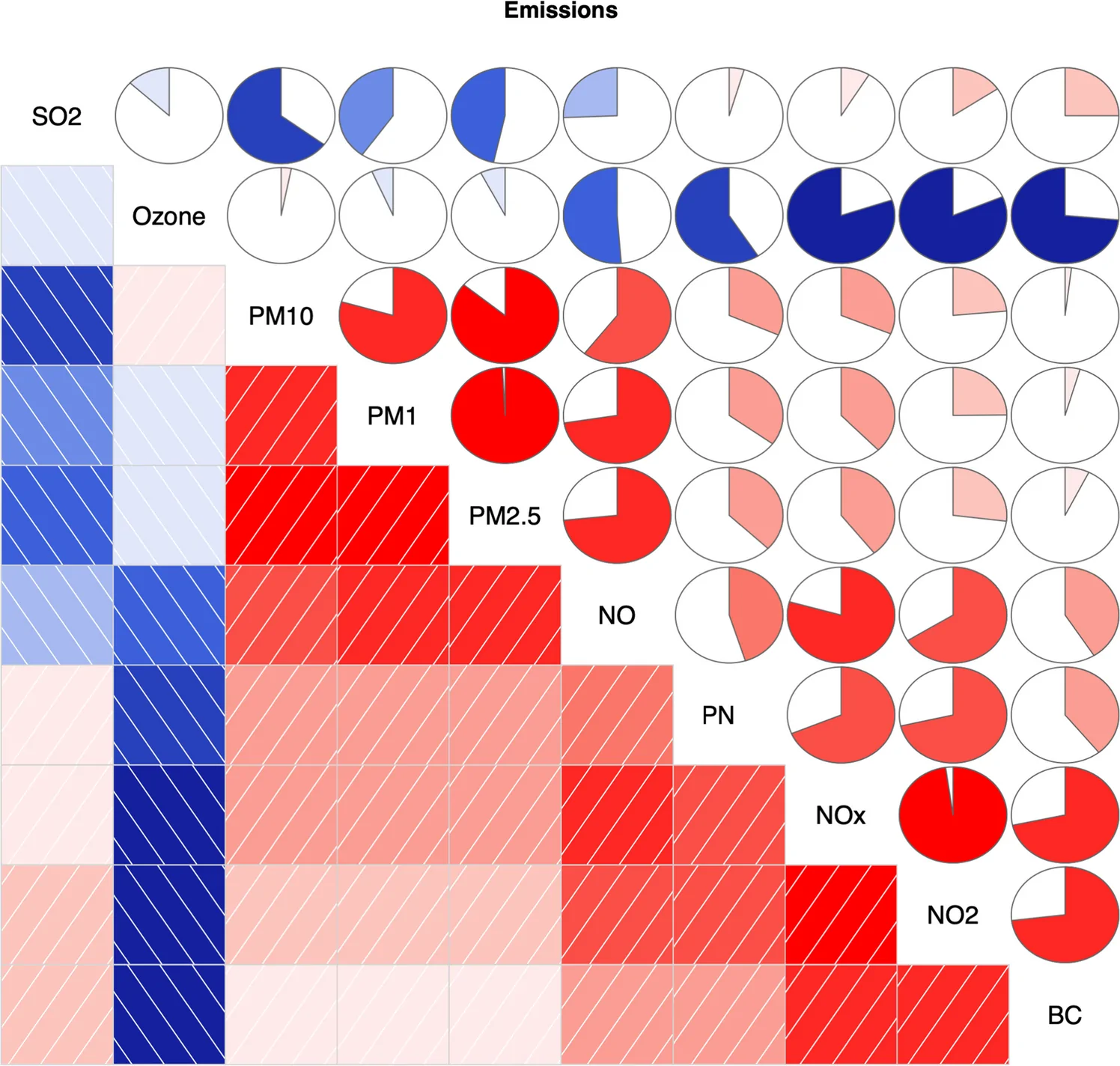
Correlogram illustrating correlations between different air quality parameters. The red and blue sectors represent positive and negative correlations, respectively. The graphs above the diagonal display Pearson’s correlation coefficients (*R*-values), where red indicates a positive correlation and blue indicates a negative correlation. The corresponding numerical values are presented in supporting information Table SI3

The correlogram in Fig. 2 illustrates the relationship between air pollutant concentrations. Numerical values and significance levels are presented in the supporting information Table SI3. The analysis shows a direct correlation between the three PM size fractions and NP concentrations. Similarly, strong correlations are observed among the three nitrogen oxides (NO, NO_2_, NOx) and BC concentrations (Fig. 2). Additionally, the results show a strong inverse correlation between O_3_ and BC, as well as with NO_2_ and NOx (Fig. 2).

### Correlation between air pollutant respiratory toxicity and air quality parameters

**Fig. 3:**
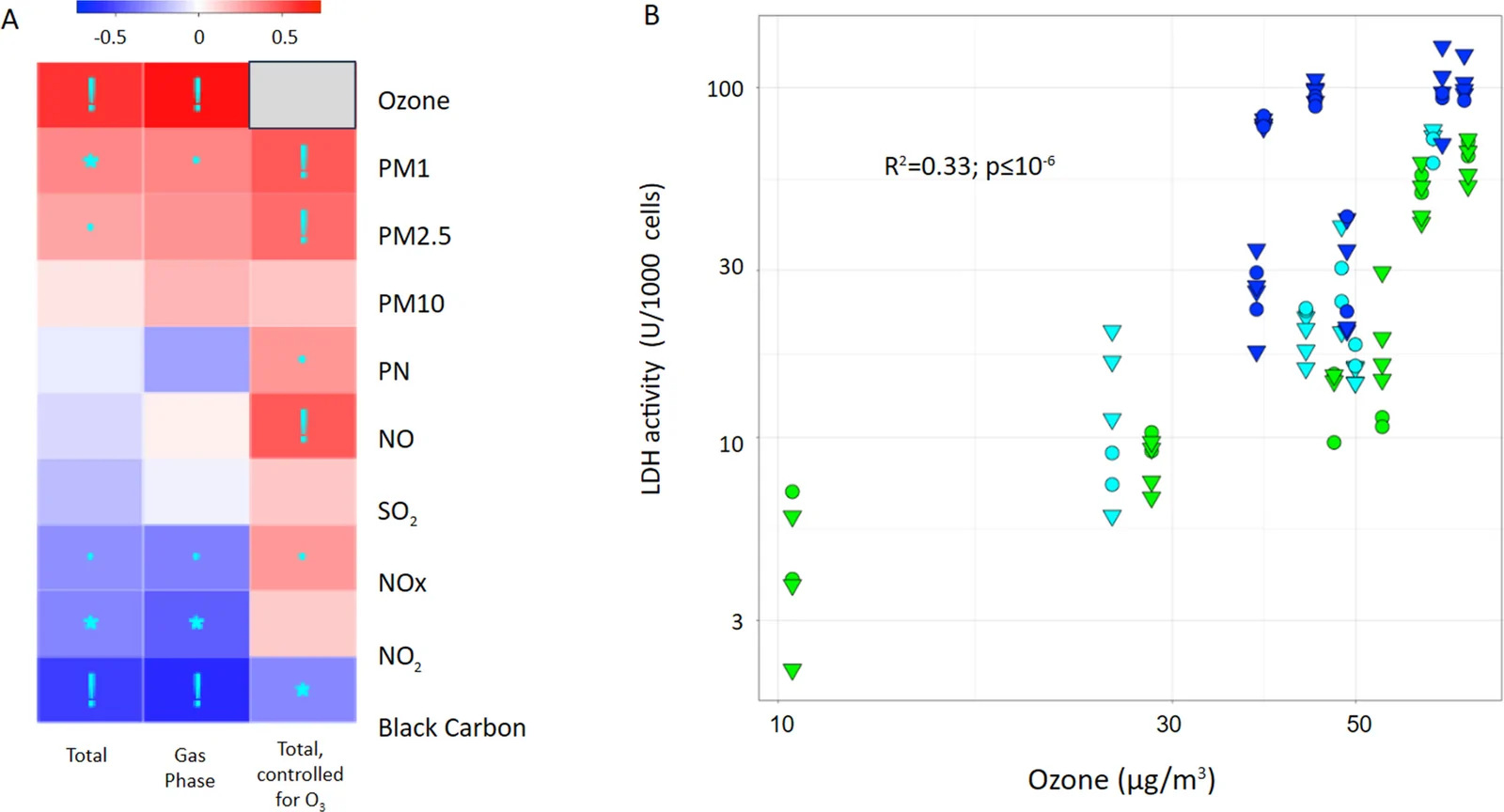
Correlation analysis between LDH activity values and air quality parameters. **A** Pearson’s correlation matrix. Red and blue sectors correspond to positive and negative correlations, respectively. Significant levels are indicated as follows: dot (**·**) *p* ≤ 0.05, star (*) *p* ≤ 0.01, exclamation mark (!) *p* ≤ 0.001). **B** Double-log correlation between O_3_ concentrations and LDH activity for different samples. Each point represents a single biological replicate. Colors correspond to the different campaigns (green for urban background, light blue for the 1st harbor campaign, and dark blue for the 2nd harbor campaign), while circles and inverted triangles correspond to unfiltered (particle + gas phases, T) and filtered (gas phase, G) samples, respectively. The coefficient of determination (*R*^*2*^) for the combined dataset is also shown

Pearson’s correlation analysis revealed a significant connection between LDH activity, as a measure of cell viability, and various air quality parameters (as measured simultaneously with the 2-h outdoor toxicity tests at the harbor and background sites). This analysis showed a strong correlation between air pollution respiratory toxicity and O_3_ concentrations in cells exposed to filtered (gases, G) or unfiltered total aerosol (particles and gases, T) (*p* ≤ 0.01, Fig. 3A). The direct relationship between toxicity and O_3_ exposure is displayed in Fig. 3B, where a double logarithmic plot shows a highly significant correlation between O_3_ concentrations and the total LDH activity (*R*^2^ = 0.33, *p* ≤ 10^−6^). The graph also illustrates that samples from the three campaigns (indicated by different symbol colors) contributed similarly to the overall regression, emphasizing that the actual pollution concentrations of each sample are more important than their location (harbor vs. background). In addition to O_3_, other factors such as particle concentrations of different size fractions (PM_1_, PM_2.5_, NP) and NO concentrations also contributed significantly to the overall respiratory toxicity of the air samples. This was evidenced through a partial correlation analysis after removal of the influence of O_3_ concentrations (Fig. 3A, last column).

**Fig. 4:**
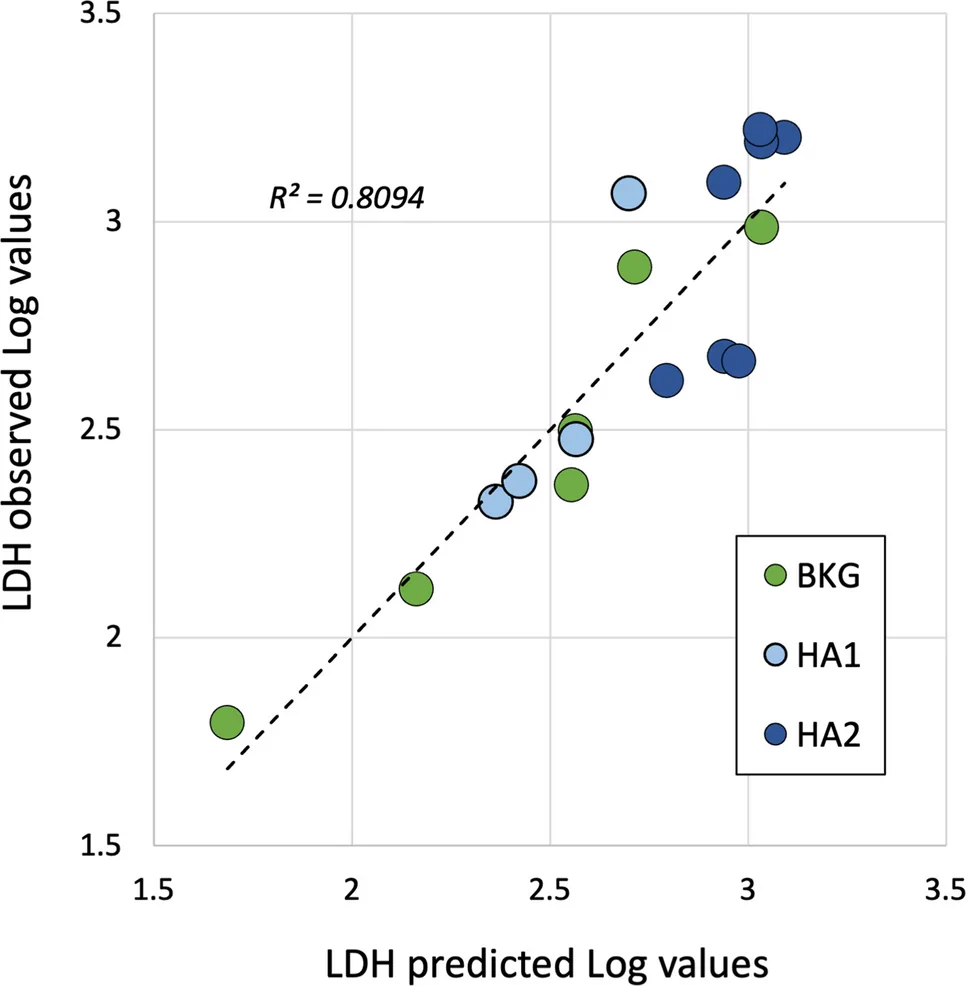
Prediction of LDH activity from air pollutant concentrations using PLS analysis. Scatter plot comparing predicted (X-axis) and observed (Y-axis) averaged LDH activity values from each sampling (log transformation). Colors correspond to the different campaigns; green for urban background, light blue for the 1st harbor campaign, and dark blue for the 2nd harbor campaign. The correlation coefficient (*R*^*2*^) is shown in the graph

The prediction of LDH activity values from air pollutant concentrations using PLS analysis (with log-transformed mean values for each sampling, only unfiltered total samples considered) identified three components (leave-one-out validation, see PLS section in the Supplementary material) that together accounted for up to 80% of LDH variance (Fig. 4). The main component, C1, explains 55.5% of the total variance and shows a strong positive contribution from O_3_ and a negative contribution from BC concentrations (Table 1, brown and blue cell colors correspond to positive and negative correlations). This component aligns with the univariate correlation analysis in Fig. 3, further supporting O_3_ as the main respiratory toxicant in the air samples. The remaining two components, C2 and C3, explain an additional 12.4% and 13.1% of the total LDH variance, respectively (note that in PLS, the order of components does not necessarily reflect their relative importance in explaining LDH variance). Component C2 is dominated by PM and NO concentrations, with additional minor contributions from NO_x_, NO_2_, and PN. In contrast, component C3 shows a strong positive contribution from SO_2_ (Table 1). The prediction accuracy, shown in Fig. 4, was consistent across the three campaigns, regardless of the sampling site (BKG *vs.* HA).

**Table 1:**
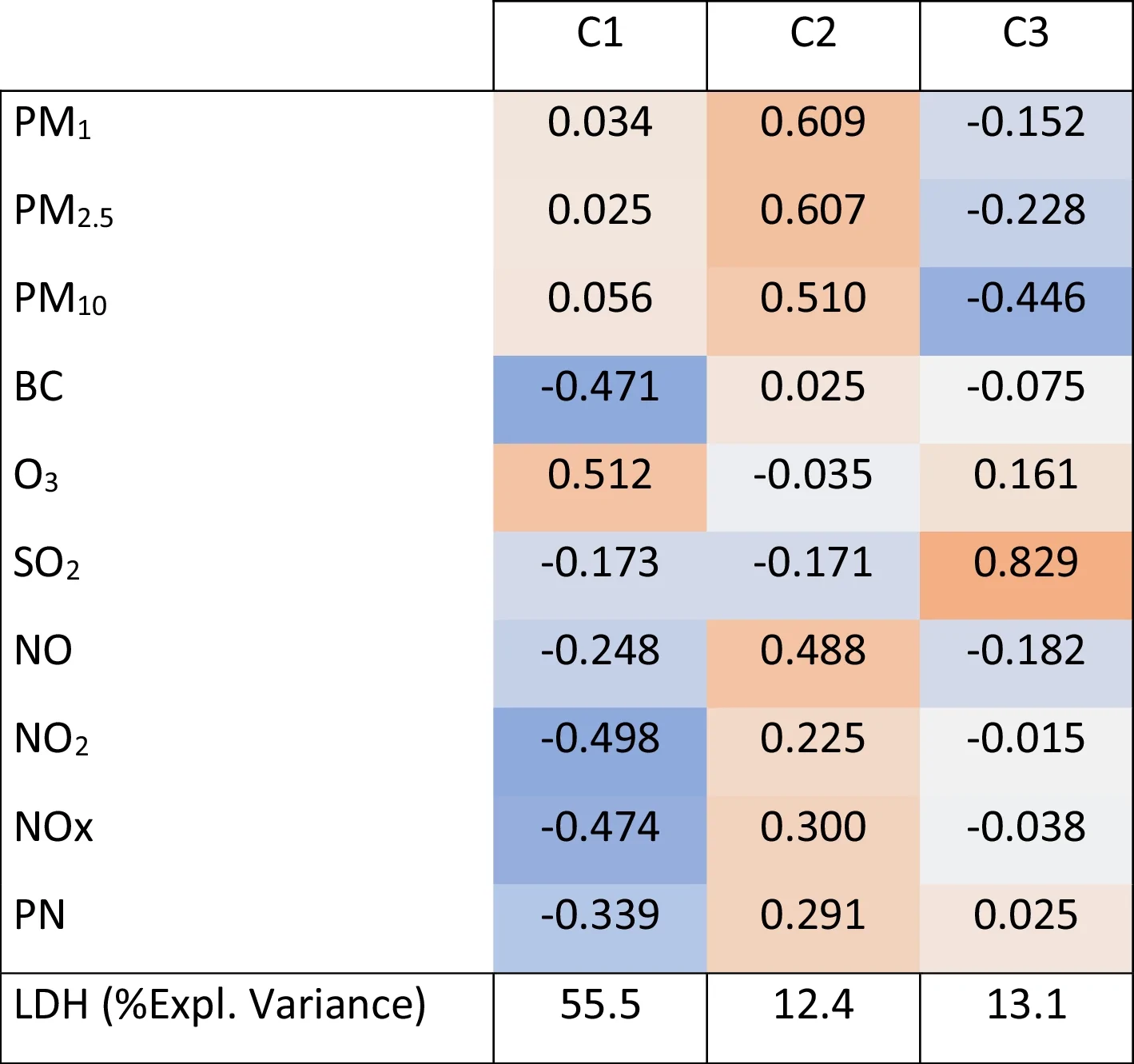
Partial least square regression results. Loadings of the different air pollutants (predictor variables) across the three components, C1, C2, and C3. The percentage of variance in the LDH activity values (predicted variable) explained by each component is shown at the bottom of the table. Cells are color-coded to indicate variables with the highest loading values (positive in orange and negative in blue) for each component

## Discussion

Air pollution is a complex and ubiquitous mixture of particles and gases that constitute significant challenges for toxicological evaluations (Mannucci et al. 2015; Portugal et al. 2024). One of the main difficulties in this regard is developing in vitro exposure systems that accurately replicate exposure by inhalation. Conventional methods generally involve collecting particles on filters, removing them from these filters, and exposing cultured cells to their extracts or suspensions. Instead, the ALI exposure system allows for direct in situ exposure, closely mimicking the inhalation of airborne particles. This approach enables exposure to air pollutant mixtures identical with those inhaled by humans, without altering the physical and chemical properties of the aerosol. In this study, we used a mobile ALI system (Juárez-Facio et al. 2024) to test toxicity of air pollutants directly in real environmental settings. Our system enables testing of the respiratory toxicity of both particulate and gaseous pollutants. There are very few reports on direct in situ cell exposure to environmental air and even fewer that also have evaluated both particulate and gas-phase toxicity (Gualtieri et al. 2018; Ding et al. 2019; Augustin et al. 2020; Zimmermann et al. 2025; Introna et al. 2025). In contrast with our current approach, none of these previous studies examined spatial and temporal aspects, nor did they prioritize identifying the most toxic components in the air pollution mixture. Therefore, we consider that the study presented here, combining ALI toxicity results and air quality data, is particularly relevant in identifying the causes and magnitude of respiratory toxicity in urban atmospheric environments.

The use of ALI systems entails certain limitations. The most important one is the relatively short exposure duration (2 h in our setup), which is required to ensure that any observed decrease in cell viability is due to ambient air exposure rather than to confounding stress factors within the ALI chambers, such as suboptimal pH control or nutrient depletion. This short exposure time limits the extent of the toxic effects observed, as the total accumulated dose reaching the cells remains relatively low. Consequently, some cell proliferation assays like the routinely utilized Trypan Blue or AlamarBlue (Resazurin) are often not sensitive enough to detect the toxic effects (Zimmermann et al. 2025). We used the lactate dehydrogenase (LDH) release assay to overcome this limitation to evaluate proliferation and viability. LDH is released from dying cells into the medium when the plasma membrane is damaged, a key feature of cell death through apoptosis or necrosis (Kumar et al. 2018). This method has previously proved particularly useful when the ratio of cell death or metabolic inhibition was low (less than 10–20%), as it provides a positive signal (live cells release negligible amounts of LDH), and it is suitable for microplate-based setups (Diamantino et al. 2001).

We used the mobile ALI system to monitor hourly variations in toxicity, enabling us to compare the respiratory toxicity of air pollutants at two well-defined sites in Barcelona: a harbor (HA) and an urban background (BKG) site. The use of appropriate filters enabled the separate assessment of gas-phase respiratory toxicity from the total aerosol exposure. In addition, we tested the influence of particle size by applying two cut-off levels (NP and PM_2.5_) in different exposure experiments conducted within the same campaign. The results revealed that toxicity levels were comparable between filtered (G) and unfiltered samples (T), regardless of the particle size. This suggests that much of the overall air pollution respiratory toxicity in the samples is associated with the gas phase.

Correlation analyses between respiratory toxicity (measured as LDH release) and air quality parameters identified O_3_ as the pollutant most strongly associated with toxicity in the studied sample set, even in non-filtered samples. A similar result was observed in a laboratory ALI study, where A549 cells were exposed to ozone, polystyrene particles, and their combination. Following exposure to ozone and ozone particle mixtures, an increased LDH release was observed, suggesting a cytotoxic effect driven by O_3_ (Henriquez et al. 2025). In our study, PLS analysis revealed that up to 55.5% of the total detected toxicity may be explained by a single component, primarily driven by high O_3_ concentrations (positive contribution) and low primary combustion emissions (NOx, BC, negative contributions). Although individual PLS components cannot be considered as direct evidence for correlation (and even less as diagnostic for a cause-effect relationship), we consider the combination of all statistical analyses (direct correlation, partial correlation, and PLS) as a strong indication for a major role of ozone in the observed cellular toxicity. Ozone is a highly irritating secondary air pollutant, which is formed from gaseous precursors (NOx and volatile organic compounds (VOCs)) under solar irradiation (Brauer et al. 2016; Donzelli and Suarez-Varela 2024). Therefore, the balance between gas- and particle-associated toxic effects may vary in regions or climates with less solar exposure. After accounting for the effect of O_3_, either through partial correlation analysis or PLS techniques, additional correlations emerged between the remaining unexplained toxicity and different air quality parameters. The parameters that significantly contributed to this residual respiratory toxicity (beyond O_3_-driven effects) included both gaseous pollutants (SO_2_, NO) and particles (predominantly PM). Together, these particles and gases (including O_3_) explained more than 80% of the total variation in respiratory toxicity, aligning closely with conventional air quality measurements and highlighting the capability of the ALI system to evaluate the toxic effects of environmental air samples. However, it should be noted that correlation does not necessarily imply causation, and these associations may reflect the influence of other co-varying pollutants that were not measured in this study. For example, tropospheric O_3_ can be associated with oxygenated VOCs, carbonyl compounds, and other secondary organic pollutants. It is therefore difficult to distinguish the direct toxic effect of ozone from the role of other compounds linked to photochemical activity.

Toxicity data reflected the impact of local emissions, with HA exposure campaigns generally showing higher respiratory toxicity than BKG air. However, due to the high contribution of O_3_, the differences were less pronounced than expected, despite the sharp contrast between the two environments. One was a relatively quiet urban location situated approximately 100 m above sea level and separated from major city roads, while the other one was a highly active harbor with emissions from ships, cars, and trucks, along with the additional impact of heavy fuel oil combustion from ships (Oeder et al. 2015; Sorte et al. 2020). The regional origin of O_3_ in the study area, and the higher consumption of O_3_ by titration (NO + O_3_ = NO_2_ + O_2_) and ozonolysis (VOC + O_3_ = OVOCs) in the city, might soften the toxicity differences between the HA and BKG sites. The observed toxicity patterns indicate a combined toxic effect influenced by the interactions among different pollutants. Regardless of the specific emission sources, the results indicate widespread pollution across the city, which may be related to Barcelona’s unique geography. The city is located between a range of hills and the Mediterranean Sea, and its compact topography, spanning less than 10 km, facilitates both the accumulation and dispersion of air pollutants.

It is noteworthy that the integrated analysis of the combined toxic effects of organic and inorganic components in both gas and particulate phases requires direct exposure of the biological model to polluted air. In this regard, ALI exposure systems provide a unique advantage, as they are the only method that enables such an assessment without the need for collection devices or extraction procedures. This pivotal characteristic may explain the disparities observed between ALI-based results and data obtained using PM suspensions and extracts, notwithstanding their collection at the same location and time. A recent study that analyzed extracts from filters revealed clear differences in air toxicity between the harbor and the urban background sites in Barcelona, as well as a strong seasonal variation between warm and cold periods (Ricarte et al. 2025). However, those results did not consider the harmful effects of the potent atmospheric gaseous oxidants found in urban atmospheres, such as O_3_ and NOx. Concentrations of NOx, VOCs, and tropospheric O_3_ at a regional scale are closely interconnected. Formation of O_3_ starts with the oxidation of NO into NO_2_ by organic (RO_2_) and hydroperoxyl (HO_2_) oxidizing radicals. These radicals mostly arise from the oxidation of VOCs (including methane, CH_4_) and CO by the hydroxyl radical (OH). Once NO_2_ is formed, photochemical transformations break down NO_2_ into NO and activated oxygen (O*). The latter interacts with oxygen molecules (O_2_) to produce O_3_ (U.S. Environmental Protection Agency 2006). In addition to their role in O_3_ production, VOCs also serve as precursors to anthropogenic secondary organic aerosols (SOA), a major component of PM_2.5_. As stated previously, the correlation between respiratory toxicity and O_3_ might, therefore, also represent the high concentrations of other secondary pollutants formed through photochemical reactions. Furthermore, as stated above, when high O_3_ concentrations are present, VOCs are intensively oxidized, and the concentration of relatively highly toxic OVOCs, such as acetaldehyde, formaldehyde, glycolaldehyde, and other aldehydes and ketones, also increases. This may also contribute to a correlation between O_3_ and respiratory toxicity. It should be noted, however, that only inorganic gases were monitored in this study, and VOC species were not measured, which limits our ability to directly assess their contribution. The negative correlation between BC and O_3_ typically occurs because the first air pollutant is mostly locally emitted and the second is of regional origin. When advection of the growth of the boundary layer favors the increase of regional O_3_ concentrations, these dilute local BC. In any case, our findings suggest that an integrated respiratory toxicological analysis of both the gas and particulate phases is required.

The cellular respiratory toxicity observed in our study can be directly linked to transport emissions, particularly to PM and gaseous pollutants (O_3_, NO, SO_2_) and, to a lesser extent, PN. Notably, while PM is generally considered as the main driver of urban air toxicity, our findings point out the importance of gaseous pollutants under real atmospheric conditions. Using the ALI system to expose human lung cells directly to ambient air, we observed that ozone caused stronger cytotoxic effects than urban PM. This apparent discrepancy is largely methodological given that traditional toxicological studies may underestimate ozone toxicity because laboratory or filter-based samples rarely contain realistic concentrations of secondary gases, and reactive gases are poorly preserved in collection systems. In contrast, the in situ ALI approach exposes cells to both particles and gases, allowing for a direct assessment of their local respiratory effects. This would explain why ozone appeared more cytotoxic in our experiments.

These findings highlight the significant contribution of road traffic and shipping emissions to urban air pollution and point out the urgency of reducing emissions from these sources. Considerable efforts have been made to reduce pollution from vehicle fuel combustion emissions, primarily focusing on PM_2.5_, PN, and NOx. García-Marles et al. found a high impact of the implementation of diesel particle filters in diesel vehicles since 2011 (EURO5/V standards) in reducing concentrations of PN and BC in a number of European cities (Garcia-Marlès et al. 2024). While the type and potency of toxic pollutants from engine combustion may change, they are unlikely to disappear entirely through changes in fuels or combustion systems. Given the challenges in assessing the toxic potential of both old and new combustion by-products and their derivatives formed by natural atmospheric processes (Grytting et al. 2024), our results indicate that achieving a clean and safe urban environment will only be possible through the transition to a decarbonized economy, starting with the transport sector. Therefore, we believe that the impending restrictions on fuel-burning vehicles (and eventually ships), especially in urban areas, are both necessary and justified.

In conclusion, this study demonstrates that air-liquid interface exposure, in combination with real-time air quality measurements, provides a powerful tool for assessing the respiratory toxic effects of individual air pollution components in situ in outdoor environments. By synchronizing temporal toxicity data with air quality parameters, we were able to identify potential key drivers of toxicity at both a harbor and an urban background site in Barcelona. By providing real-time, site-specific toxicity data, this study offers new insights into the relative contributions of different air pollutants to urban air toxicity. Our findings indicate that, among the measured pollutants, O_3_ is the primary contributor to the observed association with respiratory toxicity, based on the statistical analyses of the ALI toxicity and air quality data. However, particulate matter and nitrogen oxides also contribute, underscoring the complex interactions between different pollutants in causing overall air pollutant respiratory toxicity. These results emphasize the need to consider both gas phase and particulate pollutants when evaluating health risks associated with urban air pollution.

## Supplementary Information

Below is the link to the electronic supplementary material.

(DOCX 6.47 MB)

## Data Availability

Data will be provided on request.

## References

[CR1] Augustin P, Billet S, Crumeyrolle S, Deboudt K, Dieudonné E, Flament P, Fourmentin M, Guilbaud S, Hanoune B, Landkocz Y, Méausoone C, Roy S, Schmitt FG, Sentchev A, Sokolov A (2020) Impact of sea breeze dynamics on atmospheric pollutants and their toxicity in industrial and urban coastal environments. Remote Sens 12:1–27. 10.3390/rs12040648

[CR2] Brauer M, Freedman G, Frostad J, Van Donkelaar A, Martin RV, Dentener F, Dingenen RV, Estep K, Amini H, Apte JS, Balakrishnan K, Barregard L, Broday D, Feigin V, Ghosh S, Hopke PK, Knibbs LD, Kokubo Y, Liu Y, Ma S, Morawska L, Sangrador JLT, Shaddick G, Anderson HR, Vos T, Forouzanfar MH, Burnett RT, Cohen A (2016) Ambient air pollution exposure estimation for the global burden of disease 2013. Environ Sci Technol 50:79–88. 10.1021/ACS.EST.5B03709 26595236

[CR3] Cavanagh JA, Trought K, Brown L, Duggan S (2009) Exploratory investigation of the chemical characteristics and relative toxicity of ambient air particulates from two New Zealand cities. Sci Total Environ 407:5007–5018. 10.1016/j.scitotenv.2009.05.020 19570565

[CR4] Diamantino TC, Almeida E, Soares AMVM, Guilhermino L (2001) Lactate dehydrogenase activity as an effect criterion in toxicity tests with *Daphnia magna* straus. Chemosphere 45:553–560. 10.1016/S0045-6535(01)00029-7 11680751

[CR5] Dilger M, Armant O, Ramme L, Mülhopt S, Sapcariu SC, Schlager C, Dilger E, Reda A, Orasche J, Schnelle-Kreis J, Conlon TM, Yildirim AÖ, Hartwig A, Zimmermann R, Hiller K, Diabaté S, Paur HR, Weiss C (2023) Systems toxicology of complex wood combustion aerosol reveals gaseous carbonyl compounds as critical constituents. Environ Int 179:108169. 10.1016/j.envint.2023.108169 37688811

[CR6] Ding J, Guo J, Wang L, Chen Y, Hu B, Li Y, Huang R, Cao J, Zhao Y, Geiser M, Miao Q, Liu Y, Chen C (2019) Cellular responses to exposure to outdoor air from the Chinese Spring Festival at the air-liquid interface. Environ Sci Technol 53:9128–9138. 10.1021/acs.est.9b00399 31268311

[CR7] Donzelli G, Suarez-Varela MM (2024) Tropospheric ozone: a critical review of the literature on emissions, exposure, and health effects. Atmosphere (Basel) 15:779. 10.3390/atmos15070779

[CR8] Fröhlich E, Salar-Behzadi S (2014) Toxicological assessment of inhaled nanoparticles: role of in vivo, ex vivo, in vitro, and in silico studies. Int J Mol Sci 15:4795–4822. 10.3390/ijms15034795 PMC3975425 24646916

[CR9] Fu J, Fang T, Gao Y, Wang T, Jia Z, Guo D, Mao H (2024) Emission characteristic, spatial distribution, and health risk of polycyclic aromatic compounds (PAHs, NPAHs, and OPAHs) from light-duty gasoline and diesel vehicles based on on-road measurements. Sci Total Environ 941:173390. 10.1016/j.scitotenv.2024.173390 38815831

[CR10] Garcia-Marlès M, Lara R, Reche C, Pérez N, Tobías A, Savadkoohi M, Beddows D, Salma I, Vörösmarty M, Weidinger T, Hueglin C, Mihalopoulos N, Grivas G, Kalkavouras P, Ondracek J, Zikova N, Niemi JV, Manninen HE, Green DC, Tremper AH, Norman M, Vratolis S, Diapouli E, Eleftheriadis K, Gómez-Moreno FJ, Alonso-Blanco E, Wiedensohler A, Weinhold K, Merkel M, Bastian S, Hoffmann B, Altug H, Petit J-E, Acharja P, Favez O, Santos SMD, Putaud J-P, Dinoi A, Contini D, Casans A, Casquero-Vera JA, Crumeyrolle S, Bourrianne E, Poppel MV, Dreesen FE, Harni S, Timonen H, Lampilahti J, Petäjä T, Pandolfi M, Hopke PK, Harrison RM, Alastuey A, Querol X (2024) Source apportionment of ultrafine particles in urban Europe. Environ Int 194:109149–109149. 10.1016/j.envint.2024.109149 39566442

[CR11] Gioda A, Beringui K, Justo EPS, Ventura LMB, Massone CG, Costa SSL, Oliveira SS, Araujo RGO, Nascimento NdeM, Severino HGS, Duyck CB, de Souza JR, Saint Pierre TD (2022) A review on atmospheric analysis focusing on public health, environmental legislation and chemical characterization. Crit Rev Anal Chem 52:1772–1794. 10.1080/10408347.2021.1919985 34092145

[CR12] Grytting VS, Skuland T, Ballangby J, Refsnes M, Låg M, Øvrevik J, Mariussen E (2024) The effects of fine particulate matter (SRM 2786) on three different 3D lung models exposed at the air-liquid interface – a comparative study. Toxicol in Vitro 98:105841. 10.1016/j.tiv.2024.105841 38729454

[CR13] Gualtieri M, Grollino MG, Consales C, Costabile F, Manigrasso M, Avino P, Aufderheide M, Cordelli E, Di Liberto L, Petralia E, Raschellà G, Stracquadanio M, Wiedensohler A, Pacchierotti F, Zanini G (2018) Is it the time to study air pollution effects under environmental conditions? A case study to support the shift of in vitro toxicology from the bench to the field. Chemosphere 207:552–564. 10.1016/j.chemosphere.2018.05.130 29843032

[CR14] Hakkarainen H, Järvinen A, Lepistö T, Salo L, Kuittinen N, Laakkonen E, Yang M, Martikainen MV, Saarikoski S, Aurela M, Barreira L, Teinilä K, Ihalainen M, Aakko-Saksa P, Timonen H, Rönkkö T, Jalava P (2023) Toxicity of exhaust emissions from high aromatic and non-aromatic diesel fuels using in vitro ALI exposure system. Sci Total Environ 890:164215. 10.1016/J.SCITOTENV.2023.164215 37230343

[CR15] Henriquez AR, Godbout-Cheliak M, Filiatreault A, Thomson EM (2025) Ozone and particulate matter co-exposure at the air-liquid interface: establishing an approach to assess pollutant interactions in vitro. Toxicol in Vitro 107:106060. 10.1016/j.tiv.2025.106060 40179993

[CR16] Introna M, Juárez-Facio AT, Vallabani NVS, Tu MH, Heikkilä P, Colombo A, Liboni V, Tsyupa B, Mancini A, Keskinen J, Olofsson U, Steimer SS, Karlsson HL, Elihn K (2025) Toxicity of real-world PM2.5 road tunnel emissions using a mobile air-liquid interface system and submerged exposure. Environ Pollut 379:126486. 10.1016/j.envpol.2025.126486 40403919

[CR17] Juárez-Facio AT, Introna M, Gustavsson SÅ, Attergren M, Lindwall M, Olofsson U, Steimer SS, Elihn K (2024) A mobile air-liquid interface exposure system optimized for outdoor toxicity testing of aerosols. Aerosol Sci Technol 59:214–226. 10.1080/02786826.2024.2426487

[CR18] Kelly FJ, Fussell JC (2020) Toxicity of airborne particles—established evidence, knowledge gaps and emerging areas of importance. Philos Trans R Soc A 378:1–15. 10.1098/RSTA.2019.0322 PMC7536031 32981440

[CR19] Kim JS, Peters TM, O’Shaughnessy PT, Adamcakova-Dodd A, Thorne PS (2013) Validation of an in vitro exposure system for toxicity assessment of air-delivered nanomaterials. Toxicol in Vitro 27:164–173. 10.1016/j.tiv.2012.08.030 PMC3950355 22981796

[CR20] Kumar S, Verma MK, Srivastava AK (2013) Ultrafine particles in urban ambient air and their health perspectives. Rev Environ Health 28:117–128. 10.1515/reveh-2013-0008 24192498

[CR21] Kumar P, Nagarajan A, Uchil PD (2018) Analysis of cell viability by the lactate dehydrogenase assay. Cold Spring Harb Protoc 6:465–469. 10.1101/pdb.prot095497 29858337

[CR22] Kwon HS, Ryu MH, Carlsten C (2020) Ultrafine particles: unique physicochemical properties relevant to health and disease. Exp Mol Med 52:318–328. 10.1038/s12276-020-0405-1 PMC7156720 32203103

[CR23] Mannucci PM, Harari S, Martinelli I, Franchini M (2015) Effects on health of air pollution: a narrative review. Intern Emerg Med 10:657–662. 10.1007/s11739-015-1276-7 26134027

[CR24] Nováková Z, Novák J, Bittner M, Čupr P, Přibylová P, Kukučka P, Smutná M, Escher BI, Demirtepe H, Miralles-Marco A, Hilscherová K (2022) Toxicity to bronchial cells and endocrine disruptive potentials of indoor air and dust extracts and their association with multiple chemical classes. J Hazard Mater 424:127306. 10.1016/j.jhazmat.2021.127306 34879546

[CR25] Oeder S, Kanashova T, Sippula O, Sapcariu SC, Streibel T, Arteaga-Salas JM, Passig J, Dilger M, Paur HR, Schlager C, Mülhopt S, Diabaté S, Weiss C, Stengel B, Rabe R, Harndorf H, Torvela T, Jokiniemi JK, Hirvonen MR, Schmidt-Weber C, Traidl-Hoffmann C, BéruBé KA, Wlodarczyk AJ, Prytherch Z, Michalke B, Krebs T, Prévôt ASH, Kelbg M, Tiggesbäumker J, Karg E, Jakobi G, Scholtes S, Schnelle-Kreis J, Lintelmann J, Matuschek G, Sklorz M, Klingbeil S, Orasche J, Richthammer P, Müller L, Elsasser M, Reda A, Gröger T, Weggler B, Schwemer T, Czech H, Rüger CP, Abbaszade G, Radischat C, Hiller K, Buters JTM, Dittmar G, Zimmermann R (2015) Particulate matter from both heavy fuel oil and diesel fuel shipping emissions show strong biological effects on human lung cells at realistic and comparable in vitro exposure conditions. PLoS One 10:e0126536. 10.1371/JOURNAL.PONE.0126536 PMC4454644 26039251

[CR26] Portugal J, Bedia C, Amato F, Juárez-Facio AT, Stamatiou R, Lazou A, Campiglio CE, Elihn K, Piña B (2024) Toxicity of airborne nanoparticles: facts and challenges. Environ Int 190:108889–108889. 10.1016/j.envint.2024.108889 39042967

[CR27] Ricarte M, Portugal J, Amato F, Van Drooge BL, Jaén C, Pyambri M, Ridolfo S, Casado M, Bedia C, Elihn K, Olofsson U, Piña B (2025) Toxicity assessment of airborne ultrafine particles: role of transport emissions. Sci Total Environ 963:178435. 10.1016/j.scitotenv.2025.178435 39827641

[CR28] Rothen-Rutishauser B, Gibb M, He R, Petri-Fink A, Sayes CM (2023) Human lung cell models to study aerosol delivery - considerations for model design and development. Eur J Pharm Sci off J Eur Fed Pharm Sci 180:106337. 10.1016/J.EJPS.2022.106337 36410570

[CR29] Schraufnagel DE (2020) The health effects of ultrafine particles. Exp Mol Med 52:311–317. 10.1038/s12276-020-0403-3 PMC7156741 32203102

[CR30] Sheesley RJ, Schauer JJ, Hemming JD, Barman MA, Geis SW, Tortorelli JJ (2004) Toxicity of ambient atmospheric particulate matter from the Lake Michigan (USA) airshed to aquatic organisms. Environ Toxicol Chem 23:133–140. 10.1897/02-636 14768877

[CR31] Sorte S, Rodrigues V, Borrego C, Monteiro A (2020) Impact of harbour activities on local air quality: a review. Environ Pollut 257:113542. 10.1016/j.envpol.2019.113542 31733971

[CR32] Steenhof M, Gosens I, Strak M, Godri KJ, Hoek G, Cassee FR, Mudway IS, Kelly FJ, Harrison RM, Lebret E, Brunekreef B, Janssen NA, Pieters RH (2011) *In vitro* toxicity of particulate matter (PM) collected at different sites in the Netherlands is associated with PM composition, size fraction and oxidative potential - the RAPTES project. Part Fibre Toxicol 8:26. 10.1186/1743-8977-8-26 PMC3180259 21888644

[CR33] U.S. Environmental Protection Agency (2006) Air quality criteria for ozone and related photochemical oxidants (Final Report). U.S. Environmental Protection Agency, Washington, DC. EPA/600/R-05/004aF–cF. Available at: https://assessments.epa.gov/isa/document/&deid=149923

[CR34] Upadhyay S, Palmberg L (2018) Air-liquid interface: relevant *in vitro* models for investigating air pollutant-induced pulmonary toxicity. Toxicol Sci 164:21–30. 10.1093/toxsci/kfy053 29534242

[CR35] WHO Regional Office for Europe (2013) Review of evidence on health aspects of air pollution – REVIHAAP Project: Technical Report. WHO Regional Office for Europe, Copenhagen. Available at: https://www.who.int/europe/publications/i/item/WHO-EURO-2013-4101-43860-61757 27195369

[CR36] Zavala J, Freedman AN, Szilagyi JT, Jaspers I, Wambaugh JF, Higuchi M, Rager JE (2020) New approach methods to evaluate health risks of air pollutants: critical design considerations for in vitro exposure testing. Int J Environ Res Public Health 17:2124. 10.3390/ijerph17062124 PMC7143849 32210027

[CR37] Zimmermann EJ, Das A, Huber A, Gawlitta N, Kuhn E, Schlager C, Gutmann B, Krebs T, Schnelle-Kreis J, Delaval MN, Zimmermann R (2025) Toxicological effects of long-term continuous exposure to ambient air on human bronchial epithelial Calu-3 cells exposed at the air-liquid interface. Environ Res 269:120759. 10.1016/j.envres.2025.120759 39755196

