# Supplementary material for: Assessment of air pollution toxicity in urban environments in Barcelona, Spain, using a mobile air-liquid interface system
Source: Environ Sci Pollut Res Int. 2026 Oct 21;33(38):17633–44. doi: 10.1007/s11356-026-38306-6 (PMC13740061; doi:10.1007/s11356-026-38306-6)
Supplement: Supplementary file 1 [file 11356_2026_38306_MOESM1_ESM.docx]

**Supporting Information: Environmental Science and Pollution Research**

**Assessment of air pollution toxicity in urban environments in Barcelona, Spain, using a mobile Air-Liquid Interface system**

Fulvio Amato^1^, Maryam Pyambri^1^, Sharon Ridolfo^1^, Carmen Bedia^1^, Marina Ricarte^1^, Angeliki Karanasiou^1^, Ainhoa Rodriguez^1^, Xavier Querol^1^, Andrés Alastuey^1^, Melissa Faria^1^, Sylvia Mansilla^1^, Micol Introna^2^, Ana T. Juárez-Facio^2^, Sarah S. Steimer^2^, José Portugal^1^, Benjamin Piña^1^, Karine Elihn^2,*^

1. Institute of Environmental Assessment and Water Research, CSIC, 08034 Barcelona, Spain
2. Department of Environmental Science, Stockholm University, 11418 Stockholm, Sweden

*Corresponding author: [Karine.Elihn@aces.su.se](mailto:Karine.Elihn@aces.su.se)

Supporting Information Table SI1. Campaign description including number of samples.

| Campaign | Measurement site | Cut-off | Date | Samples |
| --- | --- | --- | --- | --- |
| 1 | Urban background | 2.5 µm | 29/09/2022 | 2F, 2P |
| 1 | Urban background | NP | 04/10/2022 | 4F, 8P |
| 1 | Urban background | NP | 05/10/2022 | 2F, 3P |
| 1 | Urban background | 2.5 µm | 06/10/2022 | 4F, 8P |
| 2 | Harbor | 2.5 µm | 24/01/2023 | 2F, 4P |
| 2 | Harbor | NP | 25/01/2023 | 4F, 6P |
| 2 | Harbor | 2.5 µm | 26/01/2023 | 4F, 6P |
| 3 | Harbor | NP | 31/01/2023 | 4F, 8P |
| 3 | Harbor | 2.5 µm | 01/02/2023 | 4F, 8P |
| 3 | Harbor | NP | 02/02/2023 | 4F, 8P |
| Totally | 95 samples (34 F, 61P) |  |  |  |

Supporting Information Table SI2. Summary of air quality data measured during ALI exposure experiments. Color-coded cells indicate relatively high (orange) and low (green) air pollutant concentrations. Averaged LDH values (last row, in blue) have been added for dataset completion

|  | Unit | UB | | | | | | HA1 | | | | | HA2 | | | | | |
| --- | --- | --- | --- | --- | --- | --- | --- | --- | --- | --- | --- | --- | --- | --- | --- | --- | --- | --- |
| Sample_Ind |  | B03 | B04 | B05 | B06 | B07 | B08 | E02 | E03 | E04 | E05 | E06 | E07 | E08 | E09 | E10 | E11 | E12 |
| FilterID |  | NDGI14 | NDGI14 | NDGI14 | NDGI14 | NDGI14 | NDGI14 | NDGI36 | NDGI36 | NDGI36 | NDGI36 | NDGI36 | NDGI38 | NDGI38 | NDGI38 | NDGI38 | NDGI38 | NDGI38 |
| Cut-off |  | 2.5 µm | UFP | UFP | UFP | 2.5 µm | 2.5 µm | 2.5 µm | UFP | UFP | 2.5 µm | 2.5 µm | UFP | UFP | 2.5 µm | 2.5 µm | UFP | UFP |
| Date |  | 29/09/2022 | 04/10/2022 | 04/10/2022 | 05/10/2022 | 06/10/2022 | 06/10/2022 | 24/01/2023 | 25/01/2023 | 25/01/2023 | 26/01/2023 | 26/01/2023 | 31/01/2023 | 31/01/2023 | 01/02/2023 | 01/02/2023 | 02/02/2023 | 02/02/2023 |
| Start time |  | 08:50:00 | 08:50:00 | 12:00:00 | 07:30:00 | 07:30:00 | 12:00:00 | 10:00:00 | 10:00:00 | 12:00:00 | 10:00:00 | 12:00:00 | 10:00:00 | 12:00:00 | 10:00:00 | 12:00:00 | 10:00:00 | 12:00:00 |
| End Time |  | 10:40:00 | 10:40:00 | 13:50:00 | 09:20:00 | 09:20:00 | 13:50:00 | 11:50:00 | 11:50:00 | 13:50:00 | 11:50:00 | 13:50:00 | 11:50:00 | 13:50:00 | 11:50:00 | 13:50:00 | 11:50:00 | 13:50:00 |
| Temperature | ºC | 18.7 | 22.8 | 25.0 | 20.0 | 20.9 | 23.9 | 8.9 | 10.6 | 12.1 | 10.7 | 12.3 | 13.2 | 14.2 | 12.4 | 13.6 | 14.3 | 14.9 |
| Relative Humidity | % | 67.5 | 74.7 | 61.7 | 90.8 | 83.4 | 79.2 | 63.1 | 60.2 | 61.3 | 59.9 | 58.3 | 46.5 | 64.5 | 42.7 | 47.0 | 51.1 | 61.2 |
| Wind speed | m/s | 2.8 | 1.0 | 2.0 | 1.5 | 2.9 | 4.6 | 1.2 | 0.7 | 1.4 | 0.7 | 1.5 | 1.2 | 1.7 | 1.4 | 1.5 | 1.3 | 1.8 |
| PN | particles/cm^3^ | 14600 | 12900 | 9800 | 21100 | 16400 | 10100 | 17600 | 15200 | 13500 | 10500 | 13400 | 16600 | 16700 | 17900 | 11597 | 16300 | 14900 |
| PM1 | µg/m^3^ | 2.1 | 5.3 | 3.0 | 7.0 | 4.0 | 6.3 | 13.1 | 8.9 | 7.8 | 10.8 | 10.8 | 15.4 | 16.6 | 14.3 | 9.0 | 20.7 | 17.4 |
| PM2.5 | µg/m^3^ | 2.6 | 6.7 | 3.8 | 8.8 | 5.0 | 7.9 | 17.5 | 14.3 | 10.4 | 12.1 | 12.5 | 17.8 | 19.1 | 16.5 | 10.6 | 24.5 | 20.3 |
| PM10 | µg/m^3^ | 7.5 | 18.4 | 10.8 | 12.8 | 11.1 | 17.5 | 65.2 | 88.8 | 35.5 | 20.1 | 23.2 | 37.6 | 39.1 | 32.6 | 20.3 | 52.2 | 40.6 |
| Black Carbon | ng/m^3^ | 335 | 1368 | 473 | 2454 | 661 | 508 | 798 | 537 | 730 | 399 | 485 | 401 | 575 | 319 | 434 | 885 | 754 |
| Ozone | µg/m^3^ | 47.1 | 28.3 | 60.1 | 10.4 | 53.8 | 68.4 | 25.4 | 49.9 | 62.0 | 43.5 | 48.1 | 38.7 | 63.7 | 44.7 | 67.7 | 37.9 | 48.8 |
| SO_2_ | µg/m^3^ | 2.7 | 2.6 | 2.9 | 2.9 | 2.4 | 2.5 | 0.2 | 0.0 | 0.3 | 0.0 | 0.8 | 0.8 | 0.7 | 0.7 | 0.6 | 0.9 | 1.2 |
| NO | µg/m^3^ | 1.6 | 10.1 | 2.7 | 20.8 | 0.9 | 1.7 | 8.2 | 8.8 | 7.6 | 5.7 | 7.4 | 14.6 | 7.3 | 10.9 | 6.7 | 17.1 | 13.4 |
| NO_2_ | µg/m^3^ | 15.3 | 42.7 | 15.5 | 52.1 | 18.6 | 9.7 | 27.4 | 21.8 | 16.6 | 12.9 | 14.5 | 27.5 | 19.0 | 24.3 | 13.0 | 34.4 | 22.8 |
| NOx | µg/m^3^ | 17.8 | 58.0 | 19.7 | 84.0 | 19.9 | 12.4 | 35.5 | 30.7 | 24.2 | 18.7 | 21.9 | 42.1 | 26.3 | 35.2 | 19.6 | 51.5 | 36.2 |
| **LDH^1^** | **U/1000 cells** | **233** | **131** | **777** | **63** | **315** | **968** | **213** | **239** | **1170** | **300** | **474** | **1241** | **1595** | **1555** | **1663** | **416** | **463** |

1) Averaged values per sampling event

Supporting information Table SI3. Correlation table between air quality parameters. Pearson’s R-values and p-values are represented below and above the diagonal, respectively. Colors indicate the strength and sense of the correlation, from strongly negative (blue) to strongly positive (red)

|  | PM_1_ | PM_2.5_ | PM_10_ | BC | O_3_ | SO_2_ | NO | NO_2_ | NOx | PN |
| --- | --- | --- | --- | --- | --- | --- | --- | --- | --- | --- |
| PM_1_ | 1.000 | 3.7E-13 | 8.9E-04 | 0.904 | 0.884 | 0.214 | 0.004 | 0.445 | 0.233 | 0.269 |
| PM_2.5_ | 0.992 | 1.000 | 1.4E-04 | 0.879 | 0.879 | 0.134 | 0.004 | 0.429 | 0.215 | 0.236 |
| PM_10_ | 0.796 | 0.860 | 1.000 | 0.942 | 0.928 | 0.014 | 0.027 | 0.466 | 0.332 | 0.332 |
| BC | 0.045 | 0.073 | 0.019 | 1.000 | 0.004 | 0.445 | 0.209 | 0.004 | 0.005 | 0.215 |
| O_3_ | -0.065 | -0.075 | 0.030 | -0.735 | 1.000 | 0.753 | 0.084 | 0.001 | 0.001 | 0.032 |
| SO_2_ | -0.402 | -0.465 | -0.647 | 0.250 | -0.130 | 1.000 | 0.445 | 0.690 | 0.876 | 0.904 |
| NO | 0.725 | 0.734 | 0.604 | 0.410 | -0.512 | -0.256 | 1.000 | 0.012 | 8.9E-04 | 0.148 |
| NO_2_ | 0.249 | 0.270 | 0.236 | 0.731 | -0.814 | 0.155 | 0.660 | 1.000 | 2.9E-10 | 0.005 |
| NOx | 0.378 | 0.394 | 0.313 | 0.716 | -0.802 | 0.087 | 0.795 | 0.978 | 1.000 | 0.007 |
| PN | 0.737 | 0.758 | 0.670 | 0.352 | -0.464 | -0.244 | 0.920 | 0.564 | 0.686 | 1.000 |

**b**

**a**

Supporting information Figure SI1. Pictures from the measurements sites. a) Fixed atmospheric monitoring laboratory at the Urban Background (UB) site. b) The ALI system installed in the fixed atmospheric monitoring laboratory. c) Mobile laboratory in a van at the harbour (HA) site. d) The ALI system installed inside the van. e) The ALI system being prepared with cell inserts before use.

**e**

**d**

**c**

Partial Least Squares Regression Model

A PLS1 model was designed using the plsreg1 script from the plsdepot library in the R environment (Sanchez & Bertrand, 2026), using the emissions and LDH data included in the Table SI2. The results of a preliminary model using 10 components is shown below. From this model, 3 components were estimated necessary for prediction (LOO cross-validation Q2 ≥ limQ2)

Predicted Variable: LDH values (averaged by sampling event), log10 transformed (17 datapoints).

Predictive Variables: PM_1_, PM_2.5_, PM_10_, Black Carbon, Ozone, SO_2_, NO, NO_2_, NOx, PN.

Cross-validation results, six components, Leave-One-Out method

| Component | PRESS^1^ | RSS^2^ | Q2^3^ | LimQ2 | Q2cum |
| --- | --- | --- | --- | --- | --- |
| **1** | **9.0751** | **16** | **0.4328** | **0.0975** | **0.4328** |
| **2** | **5.6564** | **7.123** | **0.2059** | **0.0975** | **0.5496** |
| **3** | **3.6595** | **5.1395** | **0.288** | **0.0975** | **0.6793** |
| 4 | 3.6358 | 3.0501 | -0.192 | 0.0975 | 0.6177 |
| 5 | 3.6565 | 2.729 | -0.3399 | 0.0975 | 0.4878 |
| 6 | 3.1654 | 2.6308 | -0.2032 | 0.0975 | 0.3837 |
| 7 | 2.8083 | 2.6026 | -0.079 | 0.0975 | 0.335 |
| 8 | 2.9611 | 2.5988 | -0.1394 | 0.0975 | 0.2423 |
| 9 | 2.7056 | 2.5749 | -0.0507 | 0.0975 | 0.2039 |
| 10 | 2.3021 | 2.317 | 0.0064 | 0.0975 | 0.209 |

^1^)PRESS, predicted residual error sum of squares

^2^)RSS, residual sum of squares (RSS)

^3^)Q2, predictive squared correlation coefficient

Table of X loads

| Variable | p1 | p2 | p3 | p4 | p5 | p6 | p7 | p8 | p9 | p10 |
| --- | --- | --- | --- | --- | --- | --- | --- | --- | --- | --- |
| PM1 | 0.0342 | 0.6093 | -0.1516 | -0.3608 | -0.195 | 0.1952 | 0.1128 | -1.0874 | 0.3944 | -0.5698 |
| PM2.5 | 0.0253 | 0.6069 | -0.2278 | -0.3251 | -0.0984 | 0.1528 | 0.0007 | -0.4836 | 0.0058 | 0.6634 |
| PM10 | 0.0561 | 0.5104 | -0.4462 | 0.0405 | 0.1656 | -0.2052 | -0.5112 | 2.3149 | -0.5781 | -0.0791 |
| Black Carbon | -0.471 | 0.0249 | -0.0746 | -0.3309 | 0.1215 | 0.6369 | -0.5856 | -0.0934 | 0.071 | -0.0485 |
| Ozone | 0.512 | -0.0354 | 0.1607 | 0.2926 | 0.3524 | 0.0197 | -0.4688 | -1.2484 | 0.375 | -0.009 |
| SO2 | -0.1731 | -0.171 | 0.8288 | -0.243 | -0.1457 | 0.1044 | -0.1755 | 1.2964 | -0.3398 | 0.0205 |
| NO | -0.2485 | 0.4885 | -0.1816 | 0.656 | -0.328 | 0.3383 | 0.2657 | 0.9665 | -0.2072 | -0.1147 |
| NO2 | -0.4975 | 0.2247 | -0.0153 | 0.2484 | -0.1119 | -0.2924 | -0.2159 | -1.1097 | 0.5593 | -0.269 |
| NOx | -0.4742 | 0.3003 | -0.0377 | 0.3696 | -0.1717 | -0.0829 | -0.0685 | -0.922 | -0.0401 | 0.3741 |
| PN | -0.3388 | 0.2911 | 0.0253 | -0.3908 | 1.0837 | -0.6145 | 0.3403 | 0.0293 | -0.0271 | -0.0222 |

Explained variance (R2Xy, cumulative)

| Variable | t1 | t2 | t3 | t4 | t5 | t6 | t7 | t8 | t9 | t10 |
| --- | --- | --- | --- | --- | --- | --- | --- | --- | --- | --- |
| PM1 | 0.0038 | 0.9158 | 0.9424 | 0.9692 | 0.9762 | 0.9899 | 0.9935 | 0.999 | 0.9998 | 1 |
| PM2.5 | 0.0021 | 0.9067 | 0.9667 | 0.9885 | 0.9903 | 0.9987 | 0.9987 | 0.9997 | 0.9997 | 1 |
| PM10 | 0.0103 | 0.6502 | 0.88 | 0.8804 | 0.8854 | 0.9006 | 0.9732 | 0.9983 | 1 | 1 |
| Black Carbon | 0.7256 | 0.7271 | 0.7335 | 0.7561 | 0.7588 | 0.9047 | 0.9999 | 1 | 1 | 1 |
| Ozone | 0.8573 | 0.8604 | 0.8902 | 0.9079 | 0.9308 | 0.9309 | 0.992 | 0.9993 | 1 | 1 |
| SO2 | 0.098 | 0.1699 | 0.963 | 0.9751 | 0.9791 | 0.983 | 0.9915 | 0.9994 | 1 | 1 |
| NO | 0.2019 | 0.7879 | 0.826 | 0.9148 | 0.9346 | 0.9758 | 0.9954 | 0.9998 | 1 | 1 |
| NO2 | 0.8095 | 0.9336 | 0.9338 | 0.9466 | 0.9489 | 0.9796 | 0.9926 | 0.9983 | 1 | 1 |
| NOx | 0.7354 | 0.9569 | 0.9585 | 0.9867 | 0.9922 | 0.9946 | 0.9959 | 0.9999 | 0.9999 | 1 |
| PN | 0.3754 | 0.5835 | 0.5843 | 0.6158 | 0.8321 | 0.9678 | 1 | 1 | 1 | 1 |
| LDH | 0.5548 | 0.6788 | 0.8094 | 0.8294 | 0.8356 | 0.8373 | 0.8376 | 0.8391 | 0.8552 | 0.8711 |

Reference

Sanchez G, Bertrand F (2026). Partial Least Squares (PLS) Data Analysis Methods*.* [doi:10.32614/CRAN.package.plsdepot](https://doi.org/10.32614/CRAN.package.plsdepot). R package version 0.3.1, <https://CRAN.R-project.org/package=plsdepot>.
